# Inferred Ising model unveils potentiation of pairwise neural interactions and replay of rule-learning related neural activity

**DOI:** 10.1186/1471-2202-14-S1-P276

**Published:** 2013-07-08

**Authors:** Ulisse Ferrari, Gaia Tavoni, Francesco P Battaglia, Simona Cocco, Rémi Monasson

**Affiliations:** 1 Laboratoire de Physique Theorique, Ecole Normale Superieure, 24 rue Lhomond Paris, 75005, France; 2 Laboratoire de Physique Statistique, Ecole Normale Superieure, 24 rue Lhomond Paris, 75005, France; 3 Donders Centre for Neuroscience, Nijmegen, the Netherlands

## 

In a recent experiment [1] the prefrontal cortex activity of rats was measured using multi-electtrode recordings during the awake epoch and during the previous and subsequent slow wave sleep (SWS) periods. During the awake epoch the animal faces a task, such as following a light in a Y-shaped maze, where rule learning is rewarded with food. Through the analysis of the recorded activity by means of Principal Component Analysis, the replay of the activity during the SWS after the task was shown to occur. Here we re-analyze those data with an Ising model inference algorithm (the Selective Cluster Expansion, introduced in [2]) and we show how valuable informations can be extracted from the inferred parameters in the context of neural activity replay and neuroplasticity.

We start by binning, with a fixed bin-width of 10 ms, the recording of spiking times and by computing the set of probabilities that a single neuron is active in a single time-bin and the probabilities that a couple of neurons are active together in the same time-bin. We compute the value of the parameters of the Ising model (local fields and pairwise couplings), which allow us to reproduce those probabilities [2].

**Figure 1:**
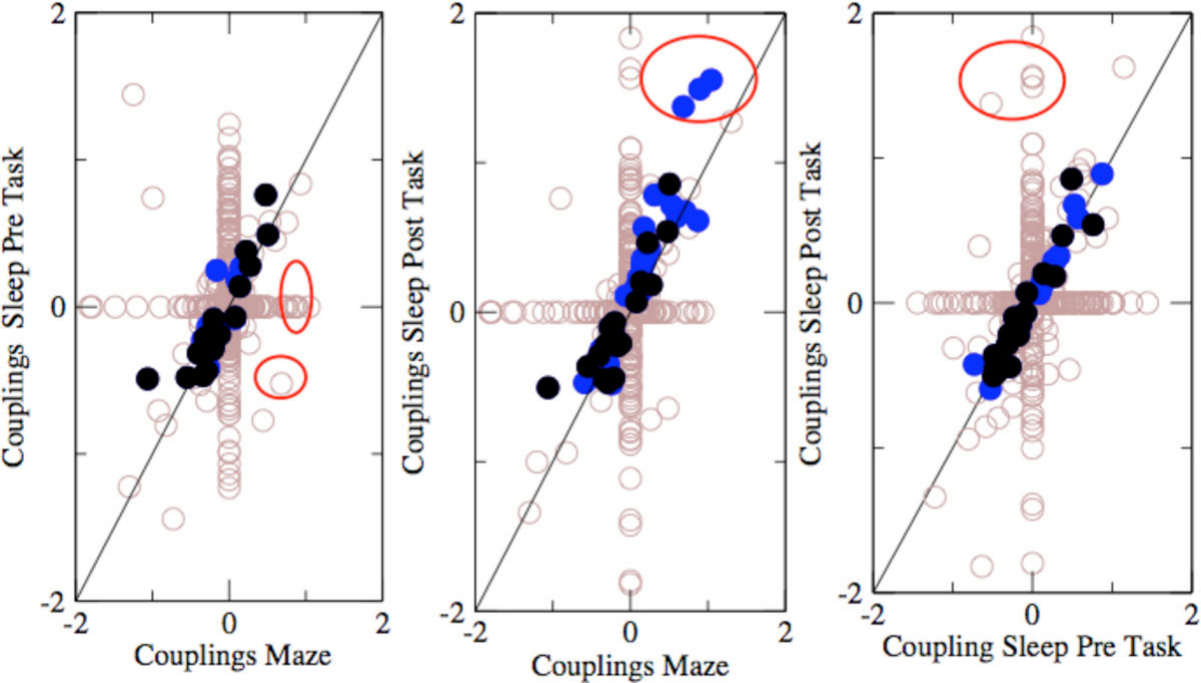
**Scattered plots of the network interactions in the three epochs**. Couplings which are statistically different from zero in the two (three) epochs are marked in blue (black). Others are marked in brow. The three couplings in the red circles, which are large and positive in the task (Maze) epoch, are potentiated in the SWS post task period with respect to the SWS pre task one. Connecting four neurons only, these couplings unveil the presence of a co-activated group of cells.

For several experimental sessions, we infer the Ising models corresponding to the recorded activity during the three epochs (SWS pre task, task, SWS post task), obtaining three different interaction networks. As shown in Figure 1 for a session in which replay was shown to occur [1] we observe generally a good agreement between the inferred networks. Moreover we unveil the presence of three strong interactions in both the task and the SWS post task epochs. These interactions, which are small in the SWS pre task period, are reinforced in the task epoch, and are even more strengthened in the SWS post task. These three interactions connect four neurons. While these four neurons are almost independent during the SWS pre task, they are strongly interacting and likely to activate each other during both the task and the SWS post task. Interestingly these strong couplings are indeed at the origin of the presence of a co-activated neural pattern created during the task and replayed during the SWS sleep post-task [3].
